# The Castration of Idiots

**Published:** 1899-07

**Authors:** 


### THE CASTRATION OF IDIOTS.

The question of the desirability of unsexing certain of the criminal classes has been given considerable attention in the editorial columns of the medical press for some time, but the possibility of preventing the multiplication of the mentally unfit seems to have received less consideration than it deserves. Any one who has taken the pains to observe the pauper classes, whether in large or small towns, is aware that its ranks are being increased from year to year, mainly by multiplication of its own kind; it is true that immigration has added to the numbers and that some degenerates have fallen into the ranks from the higher classes of society, but the greater part of the pauper tax is for the support of sons and daughters of paupers.

Reports of boards of charities show that the number of paupers is steadily increasing, and with this increase is a corresponding increase in the burden of taxation for their support. Criminals of any given class are not all totally depraved, sometimes they have been led astray by evil associations, and it is difficult to see how a law could be framed suitable for all cases, but there is no great difficulty in determining at the age of puberty whether a child has sufficient intelligence to be able to support itself. Inasmuch as the State has to provide for the vast majority of imbeciles, why should its officers not be given power to prevent the multiplication of this class?

This matter is by no means as novel as it may first seem, as is evidenced by the fact that a prominent surgeon in one of our large cities has several times been consulted with regard to this matter and has performed castration at the request of intelligent but unfortunate parents upon their imbecile children. Whilst there is an occasional imbecile child born of intelligent parents, the vast majority illustrates the law "like begets like." Not only would castration prevent procreation by these unfortunates; it would also do away with masturbation and venereal disease among them, conditions horrible for the patient and loathsome and disgusting for attendants. Nature weeds out a considerable proportion from these classes by the tedious and painful process of disease: unsexing of both males and females would be a swift and merciful way of accomplishing the same end.—*Philadelphia Med. Journal.*

